# Optimizing Post‐Acne Scar Treatment: A Pilot Comparative Study of Endo‐Radiofrequency Subcision With and Without Platelet‐Rich Plasma

**DOI:** 10.1111/jocd.70345

**Published:** 2025-07-16

**Authors:** Najmeh Ahramiyanpour, Roxana Kaveh, Elaheh Lotfi, Fatemeh Rastaghi

**Affiliations:** ^1^ Department of Dermatology Bushehr Faculty of Medicine, Bushehr University of Medical Sciences Bushehr Iran; ^2^ Guilan Road Trauma Research Center Trauma Institute, Guilan University of Medical Sciences Rasht Iran; ^3^ Yoosefabad Skin and Hair Center Tehran Iran; ^4^ Department of Dermatology Afzalipour Hospital, Afzalipour Faculty of Medicine, Kerman University of Medical Sciences Kerman Iran

**Keywords:** Endo‐RF subcision, platelet‐rich‐plasma, post‐acne scar

## Abstract

**Introduction:**

Acne is a common disorder of the pilosebaceous unit, resulting from increased sebum secretion and the colonization of *Propionibacterium*. Scarring is the most common consequence of acne and can significantly affect patients' quality of life.

**Methods:**

Patients with moderate to severe facial acne scars who were referred to the dermatology clinic in 2023 were enrolled. All patients underwent endo‐radiofrequency subcision (Endo‐RF subcision) on both sides of the face, with platelet‐rich plasma (PRP) administered on one side only. Scarring was assessed using quantitative and qualitative measures based on the Goodman and Baron's global scarring grading system.

**Results:**

Ten patients (eight females and two males) completed the study. Although qualitative scores improved in both groups, no statistically significant difference was observed. There was no significant correlation in the percentage of recovery between the two study groups (Endo‐RF and PRP + Endo‐RF) before and after treatment. Evaluations of the quantitative scores for both groups showed considerable improvements in the number of acne scars. A strongly significant correlation was observed in both groups compared to baseline. Although the combination therapy group showed greater improvement in quantitative scar scores from baseline, the difference was not statistically significant.

**Conclusion:**

Both Endo‐RF subcision and PRP combined with Endo‐RF subcision are safe and effective modalities for treating post‐acne scars. Although each method can improve acne scars, the combination therapy of Endo‐RF with PRP may yield more desirable results.

## 1 Introduction

Acne is a prevalent disorder of the pilosebaceous unit, characterized by both inflammatory and non‐inflammatory skin lesions resulting from increased sebum secretion and colonization by *Propionibacterium acnes* [1, 2]. It is estimated to be the eighth most common disease, affecting 9.3% of the population, while acne vulgaris accounts for 16% of all skin disorders. In the United States alone, 40–50 million people are affected by acne, which leads to significant economic costs [3, 4, 5]. While acne can impact individuals of any gender and age, it most commonly affects those between 12–24 years old [1, 3].

The most common consequence of acne is scarring, which is reported in approximately 95% of patients, though only 30% develop significant scarring [6]. Post‐acne scars typically result from the healing process of deep inflammatory lesions or delayed treatment of mild to moderate cases. These scars can have substantial psychosocial effects, including, lower self‐esteem, self‐satisfaction, and reduced occupational opportunities, thereby impacting the quality of life [3, 6].

Despite extensive research into treatment options such as subcision, punch excision, microneedling, and chemical peeling, an ideal treatment has yet to be established [7]. Subcision is a technique used to treat acne scars by breaking fibrotic bands, lifting depressed scars, and promoting neocollagenesis. A modification of this technique, endo‐radiofrequency (Endo‐RF) subcision, combines subcision with radiofrequency radiation. The RF component delivers controlled thermal stimulation to the dermis, which enhances fibroblast activity, promotes collagen remodeling, and supports wound healing. This combined modality may offer improved outcomes in scar elevation and texture compared to conventional subcision alone [8, 9, 10, 11, 12].

Platelet‐rich plasma (PRP) is another valuable treatment modality, containing over 20 growth factors that play a crucial role in cell differentiation and regeneration. PRP has been shown to be highly effective in skin rejuvenation, hair growth, skin regeneration, acne management, and wound healing. PRP is believed to increase collagen synthesis and modulate inflammation through the release of growth factors like PDGF, TGF‐β, and VEGF. Although the exact biological mechanisms remain incompletely understood [7, 13, 14, 15, 16, 17].

Given the potential benefits of these methods, this study aims to evaluate the efficacy of Endo‐RF subcision alone versus its combination with PRP for the treatment of post‐acne scars.

## 2 Materials and Methods

### 2.1 Study Design

The current study was designed as a split‐face, randomized clinical trial approved by the relevant institutional ethics committee. All ethical principles were adhered to according to the World Medical Association's Declaration of Helsinki.

Data regarding the study methods, treatment benefits, and potential side effects were thoroughly explained to patients before enrollment, and all participants provided written informed consent.

### 2.2 Sample Size

A pilot design was selected for this research because of the study's novel approach. The minimum required sample size was estimated to be 10 participants. The study involved patients with mild to severe facial acne scars who were referred to the dermatology clinic in 2023.

### 2.3 Participants

The study included patients aged 18 and older with moderate to severe atrophic acne scars on their faces, primarily in the malar region. Participants were chosen after undergoing a thorough physical examination by a dermatologist. Exclusion criteria included individuals with cardiac pacemakers, metal implants, active acne lesions, infections at the scar sites, pregnancy or lactation, or prior treatments such as laser therapy, subcision, or peeling within the previous 4 months.

### 2.4 Intervention

The patients' faces were cleaned using a sterile set before initiating the procedure. The intervention area, primarily the malar region, received local anesthesia through the injection of 2% lidocaine. All patients underwent Endo‐RF subcision on both sides of their faces, followed by the injection of PRP on one side during a single treatment session.

Endo‐RF subcision was performed using the Afrodite device (3MED Group) at a power setting of 30 watts and a temperature of 38°C–40°C. Subcision was done in the scar areas on both sides of the face using an 18G cannula to achieve the desired effect. In the final step, PRP was randomly injected into one side of the face.

To prepare PRP, 5 mL of the patient's venous blood was collected and centrifuged at 2500G/10 min. The buffy coat and plasma layers were then extracted and re‐centrifuged at 3500G/10 min. Centrifugation was performed using a Universal Centrifuge manufactured by Arad Teb Farzam, Iran. The obtained PRP was injected intradermally using a 30G BD insulin syringe, with 0.1 mL injected at each point. Injection points were spaced 1 cm apart across the acne‐scarred areas.

### 2.5 Outcome Assessment

Prior to commencing the study, all participants completed a demographic questionnaire, providing information on their name, age, sex, past drug history, medical history, and duration of their condition. Digital photographs were taken of the participants' scars to evaluate their number, severity, and type. Assessments were conducted at two time points: at baseline and 3 months after treatment. The Goodman and Baron grading system was employed at both evaluations for quantitative and qualitative analysis.

### 2.6 Statistical Analysis

Data were analyzed using SPSS version 26. To evaluate the significance of differences between baseline and post‐treatment values, and to compare variable distributions between groups, the Wilcoxon test was used. A *p* value of less than 0.05 was considered statistically significant.

## 3 Results

Ten patients with moderate to severe atrophic acne scars on the cheeks were evaluated during this study. Endo‐RF subcision was performed on both sides of the face for all patients, with PRP randomly administered to one side. The average age of the patients was 27.7 years. Eighty percent were female, and 20% were male.

Qualitative and quantitative grading of scars showed favorable post treatment responses for both methods. In the Endo‐RF group, baseline scar severity was moderate in three cases and severe in seven. After treatment, scar severity improved to macular in one patient, mild in five, moderate in two, and remained severe in two patients. However, this improvement was not statistically significant (*p* = 0.23). In the Endo‐RF + PRP group, baseline scar severity was moderate in four patients and severe in six. Post treatment evaluations showed scar improvement to macular in one patient, mild in six, moderate in two, and severe in one. This improvement was also not statistically significant (*p* = 0.29).

There was no significant difference in the percentage of recovery between the two study groups (Endo‐RF and Endo‐RF + PRP) before and after treatment (*p* = 0.63; *p* = 0.93) (Table 1).

**TABLE 1:** Goodman and Baron's qualitative scores before and after the intervention.

Group	Variable	Baseline	After the procedure	*p* value
Endo‐RF	Macular	0	1 (10)	
	Mild	0	5 (50)	0.23
	Moderate	3 (30)	2 (20)	
	Severe	7 (70)	2 (20)	
	Macular	0	1 (10)	
PRP +Endo‐RF	Mild	0	6 (60)	0.29
	Moderate	4 (40)	2 (20)	
		6 (60)	1 (10)	
	*p* value	0.63	0.93	

**FIGURE 1:**
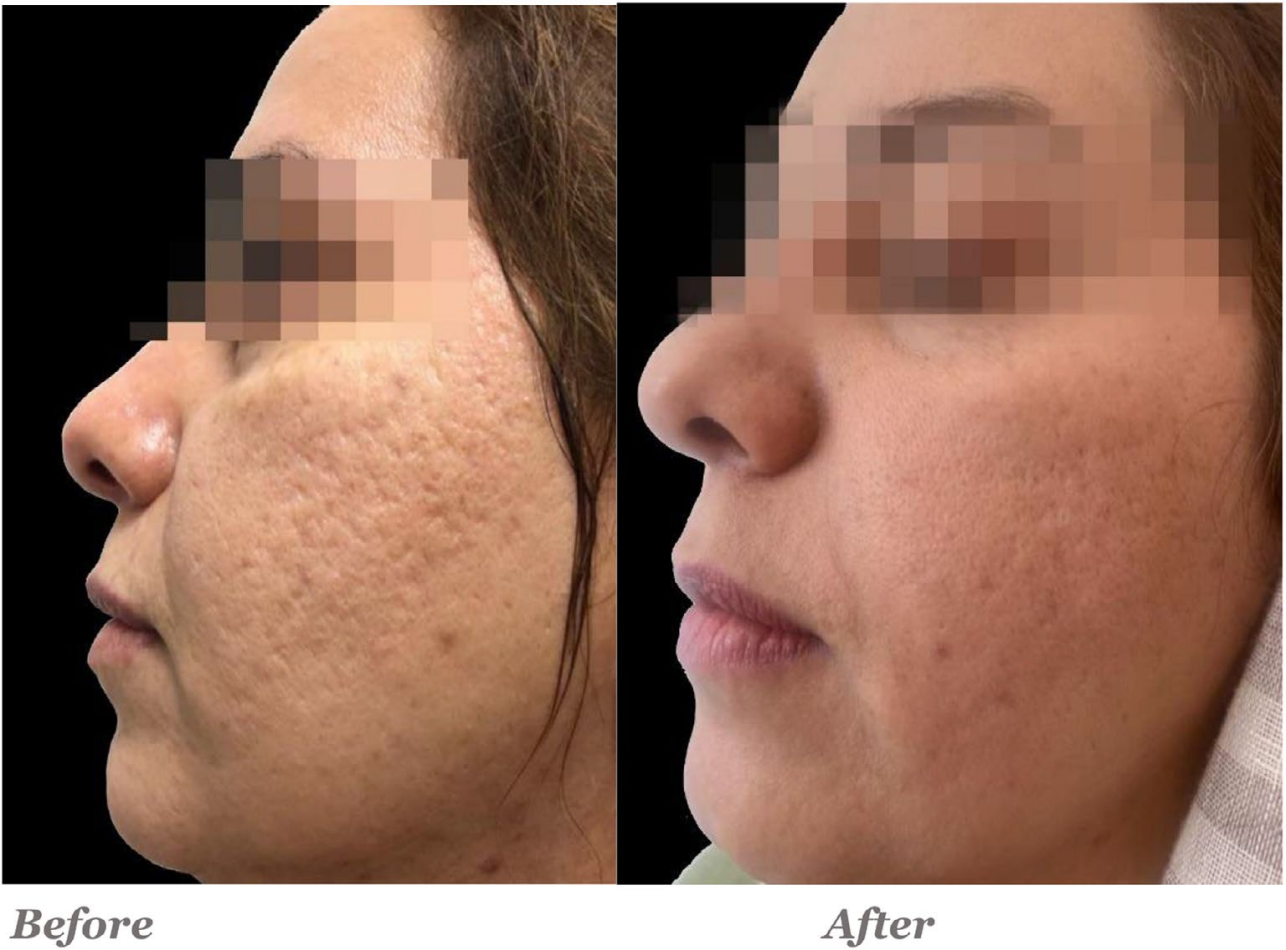
PRP+ Endo‐RF was done for the left side. Goodman and baron acne scar grading: Qualitative score: Before: Grade 3–4 scarring. After: The skin shows noticeable improvement, scars reduced to Grade 2–3. Even though there are still present, they appear shallower. Quantitative score: Before: Severe to very severe range. After: There is a significant reduction in scar visibility, bringing the count down to the moderate range. The skin appears more even, with a reduction in scar depth and overall smoother texture.

**FIGURE 2:**
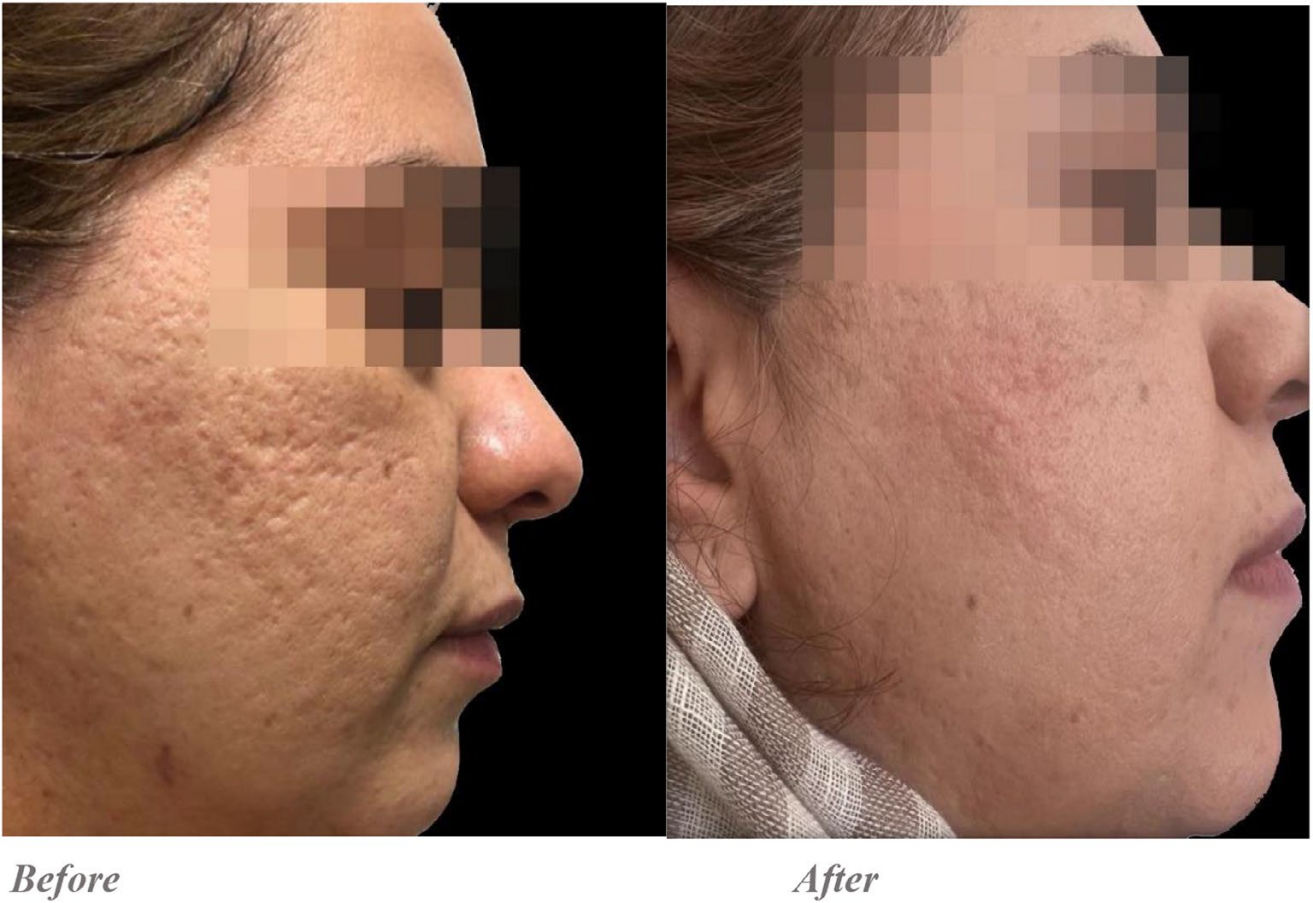
Endo‐RF was done for the right side. Goodman and baron acne scar grading: Qualitative score: Before: The skin displays Grade 3–4 acne scars. After: The skin shows a reduction in scar severity to Grade 2–3. Many scars appear shallower, with less shadowing and improved texture. Quantitative score: Before: Severe scaring with multiple deep atrophic scars contributing to an uneven skin surface. After: The number of scars appears significantly reduced, moving to the moderate category. The scars are shallower, and the skin texture is smoother.

**FIGURE 3:**
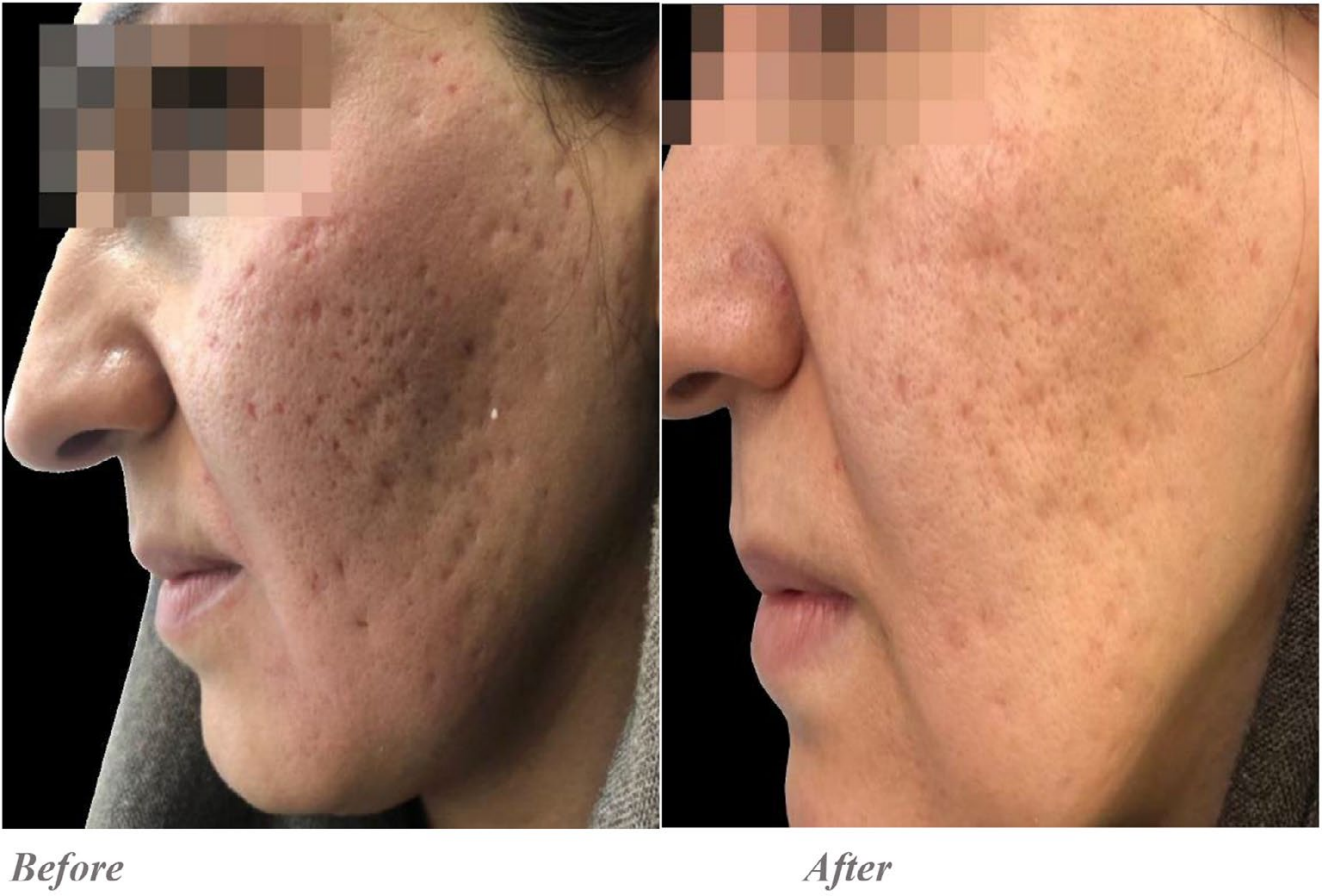
PRP+ Endo‐RF was done for the left side. According to the Goodman and Baron qualitative global scarring grading system, the patient's scars were initially classified as Grade 4 (severe), with numerous deep, visible atrophic scars. Post treatment evaluation showed marked improvement, with residual shallow scarring, corresponding to a Grade 2–3 (mild to moderate) classification.

**FIGURE 4:**
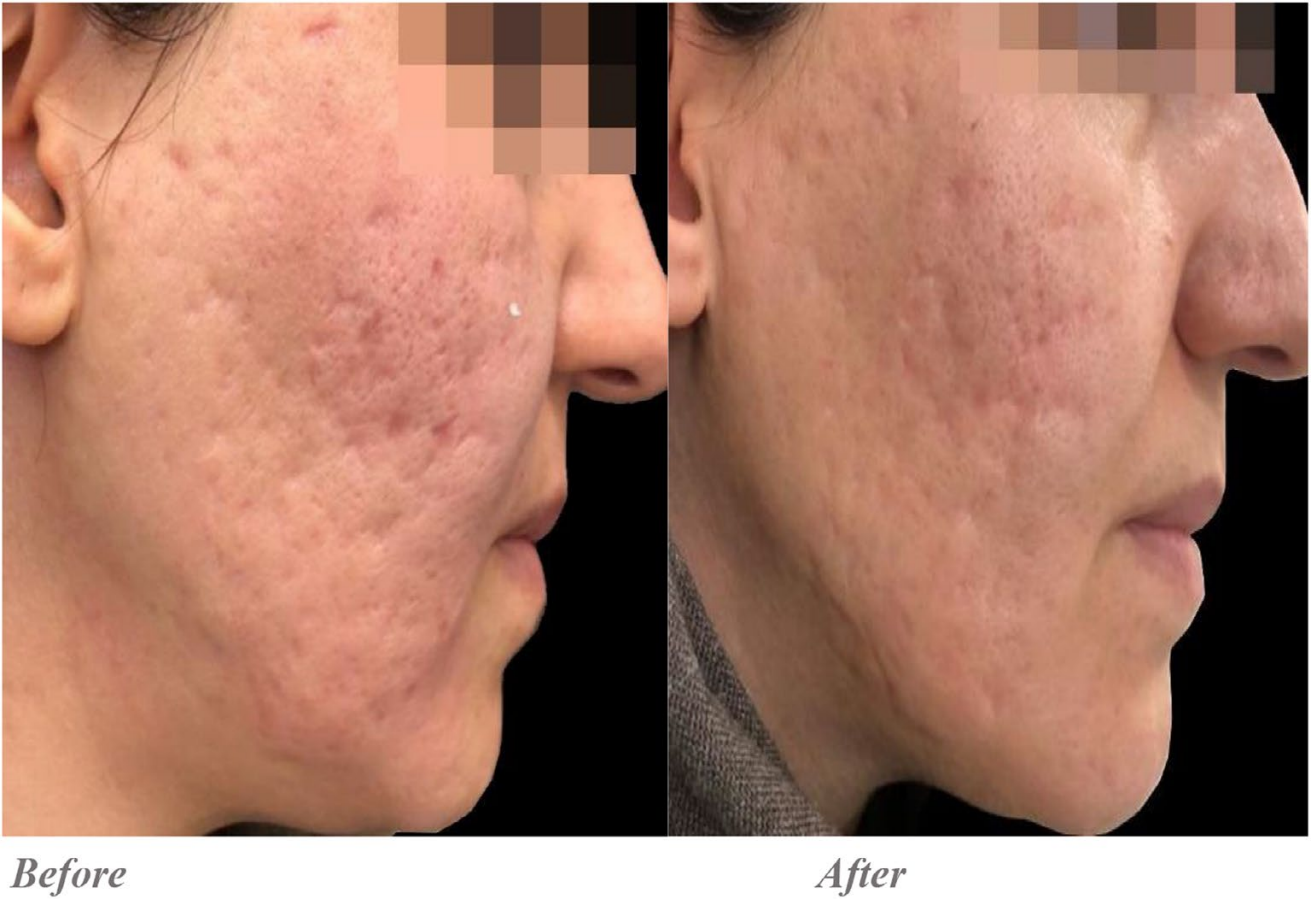
Endo‐RF was done for the right side. Based on the Goodman and Baron qualitative grading system, the scarring severity prior to treatment was assessed as Grade 4 (severe), with multiple deep, atrophic scars including icepick and boxcar types. Post treatment, the patient's scars showed a notable reduction in depth and surface irregularity, corresponding to a Grade 2–3 (mild to moderate) classification.

Quantitative evaluations showed that in the Endo‐RF group, the average score decreased from 12.5 ± 1.87 pre‐intervention to 8.8 ± 1.59 post‐intervention, with a statistically significant difference (*p* = 0.001). In the PRP + RF group, the average score improved from 12.65 ± 1.5 to 6.3 ± 0.86, and this difference was also statistically significant (*p* = 0.001) (Figures 1, 2, 3, 4).

**Graph 1:**
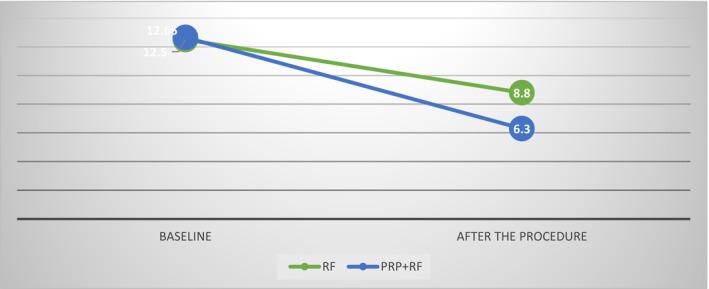
Goodman and Baron's quantitative scores before and after the intervention.

Although the combined PRP + RF group showed a greater average improvement in post treatment quantitative scores than the RF group, this difference was not statistically significant (*p* = 0.95; *p* = 0.18) (Graph 1, Table 2). No significant adverse effects were observed in either group.

**TABLE 2:** Goodman and Baron's quantitative scores before and after the intervention.

Group	Baseline	After the procedure	Mean difference	*p* value
Endo‐RF	12.5 ± 1.87	8.8 ± 1.59	3.7 ± 0.73	< 0.001
PRP + Endo‐RF	12.65 ± 1.5	6.3 ± 0.86	6.35 ± 0.81	< 0.001
*p* value	0.95	0.18		

## 4 Discussion

Scarring is the most common consequence of acne, significantly affecting patients' mental, emotional, and occupational well‐being, and directly impacting their quality of life [18]. Various modalities have been used to address this issue and improve aesthetic outcomes, including microneedling, lasers, subcision, peeling, fillers, and PRP. Studies have confirmed the benefits of microneedling in treating acne scars, with greater improvements observed when microneedling is combined with subcision compared to microneedling alone. However, studies on the combined use of PRP and microneedling are less conclusive [19, 20, 21].

Lasers are another effective modality in treating erythema, dyspigmentation, and decreased elasticity of macular acne scars. Among all, Nd‐YAG and fractional CO_2_ lasers are the most commonly used [22]. The use of PRP has increased in dermatology due to its various growth factors that promote skin rejuvenation, regeneration, and wound healing. PRP can improve atrophic acne scars when used alone or in combination with other treatments. Combining PRP with fractional CO_2_ laser treatment has shown significant improvements in scarring, shorter downtime, and fewer side effects such as edema or erythema. Both topical and intradermal applications of post‐laser PRP have demonstrated similar efficacy [7, 20, 23, 24].

Another option for treating post‐acne scars is subcision, which breaks fibrotic bands, frees dermal papillae, and induces neocollagenesis. Previous studies have shown that various subcision techniques, including needle‐based subcision, cannula‐based subcision, and Endo‐RF subcision, can significantly improve acne scarring, with no single method showing superior results [25, 26]. Endo‐RF subcision, a modification that combines subcision with radiofrequency radiation, stimulates collagen production and wound healing through electromagnetic waves, leading to scar improvement. A case series by Nilforoushzadeh et al. demonstrated significant improvement in acne scars treated with Endo‐RF subcision [8].

Our study aimed to evaluate a novel approach to treating post‐acne scarring: the combination of PRP and Endo‐RF subcision versus Endo‐RF subcision alone. Data analysis showed improvements in the number of scars and their severity in both study groups. Both quantitative and qualitative scar assessments improved compared to baseline. The combination therapy group showed greater improvement in the number of scars. These findings suggest that both methods can effectively improve post‐acne scarring, with the combination therapy showing greater quantitative benefits. Our results support previous research highlighting the efficacy of combining subcision with PRP. Deshmukh et al. reported that combination therapy of subcision and PRP led to greater improvement in acne scarring compared to subcision alone [27]. Conversely, Hassan et al. reported better results with fewer side effects using PRP monotherapy compared to PRP combined with subcision [28]. The synergistic effect of combining subcision with other modalities, as confirmed in our research, has been supported by other studies. For example, RF‐subcision combined with polycaprolactone‐based dermal fillers showed significant effects due to the collagen‐stimulating properties of polycaprolactone fillers [29]. To minimize complications and prevent re‐depression of scars, simultaneous use of platelet gel or HA gels is recommended [30, 31]. Suctioning with a microdermabrasion device can further enhance the impact of subcision on acne scarring [32]. As our study proposes Endo‐RF subcision combined with PRP as a safe and highly effective treatment for post‐acne scarring, it is important to acknowledge its limitations. One important limitation of our study is the small number of participants, which makes it more difficult to achieve strong statistical results and limits how much we can apply the findings to the general population. In addition, we did not control for factors such as skin type, scar type, acne duration, or previous treatments. These factors may have influenced the results. Another limitation is that the study did not use objective methods to check the improvement. It was based on clinical grading scales, which are standard but still subjective and may lead to differences between evaluators. We recommend that future studies include a larger number of participants and take these variables into account.

## 5 Conclusion

This study demonstrates that both Endo‐RF subcision and PRP combined with Endo‐RF subcision are safe and effective modalities for treating post‐acne scars. Although both methods can improve acne scars, the combination therapy of Endo‐RF and PRP can achieve more favorable results.

## Author Contributions

All authors contributed significantly to this study. N.A. was responsible for study design, methodology, and manuscript editing. R.K. contributed to data collectionand drafting of the manuscript. E.L. participated in data collection and draftingediting of the manuscript. F.R. oversaw project administration, data collection, interpreted the results, and performed the revision of the manuscript. All authors read and approved the final manuscript and agree to be accountable for all aspects of the work.

## Ethics Statement

The study was approved by the Ethics Committee of Kerman University of Medical Sciences, code: “IR.KMU.AH.REC.1401.207” and the IRCT code of “IRCT20221110056463N1”.

## Consent

Written informed consent was obtained from all participants for the publication of photographs included in this study. The images are anonymized and do not contain any identifiable personal information.

## Conflicts of Interest

The authors declare no conflicts of interest.

## Data Availability

The data that support the findings of this study are available from the corresponding author upon reasonable request.

## References

[1] J. L. Bolognia , J. V. Schaffer , and L. Cerroni , Dermatology, Fourth ed. (Elsevier, 2018).

[2] H. C. Williams , R. P. Dellavalle , and S. Garner , “Acne Vulgaris,” Lancet 379, no. 9813 (2012): 361–372. 21880356 10.1016/S0140-6736(11)60321-8

[3] J. K. Tan and K. Bhate , “A Global Perspective on the Epidemiology of Acne,” British Journal of Dermatology 172, no. S1 (2015): 3–12. 10.1111/bjd.13462 25597339

[4] R. J. Hay , N. E. Johns , H. C. Williams , et al., “The Global Burden of Skin Disease in 2010: An Analysis of the Prevalence and Impact of Skin Conditions,” Journal of Investigative Dermatology 134, no. 6 (2014): 1527–1534. 24166134 10.1038/jid.2013.446

[5] C. Karimkhani , R. P. Dellavalle , L. E. Coffeng , et al., “Global Skin Disease Morbidity and Mortality: An Update From the Global Burden of Disease Study 2013,” JAMA Dermatology 153, no. 5 (2017): 406–412. 28249066 10.1001/jamadermatol.2016.5538 PMC5817488

[6] S. Z. Ghodsi , H. Orawa , and C. C. Zouboulis , “Prevalence, Severity, and Severity Risk Factors of Acne in High School Pupils: A Community‐Based Study,” Journal of Investigative Dermatology 129, no. 9 (2009): 2136–2141. 19282841 10.1038/jid.2009.47

[7] H. I. Gawdat , R. A. Hegazy , M. M. Fawzy , and M. Fathy , “Autologous Platelet Rich Plasma: Topical Versus Intradermal After Fractional Ablative Carbon Dioxide Laser Treatment of Atrophic Acne Scars,” Dermatologic Surgery 40, no. 2 (2014): 152–161. 24354616 10.1111/dsu.12392

[8] M. A. Nilforoushzadeh , M. Heidari‐Kharaji , T. Fakhim , et al., “Endo‐Radiofrequency Subcision for Acne Scars Treatment: A Case Series Study,” Journal of Cosmetic Dermatology 21 (2022): 5651–5656. 35770321 10.1111/jocd.15195

[9] S. Yadav and S. Gupta , “Radiofrequency‐Assisted Subcision for Postacne Scars,” Journal of the American Academy of Dermatology 78, no. 1 (2018): e9–e10. 29241808 10.1016/j.jaad.2017.07.037

[10] D. S. Orentreich and N. Orentreich , “Subcutaneous Incisionless (Subcision) Surgery for the Correction of Depressed Scars and Wrinkles,” Dermatologic Surgery 21, no. 6 (1995): 543–549. 7773602 10.1111/j.1524-4725.1995.tb00259.x

[11] A. M. Rotunda , A. R. Bhupathy , and T. E. Rohrer , “The New Age of Acne Therapy: Light, Lasers, and Radiofrequency,” Journal of Cosmetic and Laser Therapy 6, no. 4 (2004): 191–200. 16020203 10.1080/14764170410008124

[12] D. P. Friedmann , G. L. Vick , and V. Mishra , “Cellulite: A Review With a Focus on Subcision,” Clinical, Cosmetic and Investigational Dermatology 10 (2017): 17. 28123311 10.2147/CCID.S95830 PMC5234561

[13] M. K. Shin , J. H. Lee , S. J. Lee , and N. I. Kim , “Platelet‐Rich Plasma Combined With Fractional Laser Therapy for Skin Rejuvenation,” Dermatologic Surgery 38, no. 4 (2012): 623–630. 22288389 10.1111/j.1524-4725.2011.02280.x

[14] S. Kim , H.‐W. Ryu , K.‐S. Lee , and J.‐W. Cho , “Application of Platelet‐Rich Plasma Accelerates the Wound Healing Process in Acute and Chronic Ulcers Through Rapid Migration and Upregulation of Cyclin A and CDK4 in HaCaT Cells,” Molecular Medicine Reports 7, no. 2 (2013): 476–480. 23242428 10.3892/mmr.2012.1230

[15] Z. Ebrahimi , Y. Alimohamadi , M. Janani , P. Hejazi , M. Kamali , and A. Goodarzi , “Platelet‐Rich Plasma in the Treatment of Scars, to Suggest or Not to Suggest? A Systematic Review and Meta‐Analysis,” Journal of Tissue Engineering and Regenerative Medicine 16 (2022): 875–899. 35795892 10.1002/term.3338

[16] A. G. Evans , J. M. Mwangi , R. W. Pope , et al., “Platelet‐Rich Plasma as a Therapy for Androgenic Alopecia: A Systematic Review and Meta‐Analysis,” Journal of Dermatological Treatment 33, no. 1 (2022): 498–511. 32410524 10.1080/09546634.2020.1770171

[17] H. I. Gawdat , Y. A. El‐Hadidy , R. S. Allam , and H. A. Abdelkader , “Autologous Platelet‐Rich Plasma ‘Fluid’ Versus ‘Gel’ Form in Combination With Fractional CO_2_ Laser in the Treatment of Atrophic Acne Scars: A Split‐Face Randomized Clinical Trial,” Journal of Dermatological Treatment 33 (2022): 1–2663. 35435087 10.1080/09546634.2022.2067816

[18] J. A. Halvorsen , R. S. Stern , F. Dalgard , M. Thoresen , E. Bjertness , and L. Lien , “Suicidal Ideation, Mental Health Problems, and Social Impairment Are Increased in Adolescents With Acne: A Population‐Based Study,” Journal of Investigative Dermatology 131, no. 2 (2011): 363–370. 20844551 10.1038/jid.2010.264

[19] R. Hassan , “Comparison of Efficacy of Micro Needling for the Treatment of Acne Scars in Asian Skin With and Without Subcision,” Journal of the Turkish Academy of Dermatology 9, no. 2 (2015): 68–78.

[20] M. J. Hesseler and N. Shyam , “Platelet‐Rich Plasma and Its Utility in the Treatment of Acne Scars: A Systematic Review,” Journal of the American Academy of Dermatology 80, no. 6 (2019): 1730–1745. 30742878 10.1016/j.jaad.2018.11.029

[21] M. El‐Domyati , M. Barakat , S. Awad , W. Medhat , H. El‐Fakahany , and H. Farag , “Microneedling Therapy for Atrophic Acne Scars: An Objective Evaluation,” Journal of Clinical and Aesthetic Dermatology 8, no. 7 (2015): 36–42. PMC4509584 26203319

[22] N. Sarvipour , Z. Akbari , M. Shafie'ei , M. Jamali , M. Ahmadzade , and N. Ahramiyanpour , “Lasers for the Treatment of Erythema, Dyspigmentation, and Decreased Elasticity in Macular Acne Scars: A Systematic Review,” Lasers in Medical Science 37, no. 9 (2022): 3321–3331. 35918567 10.1007/s10103-022-03621-0

[23] O. H. Alser and I. Goutos , “The Evidence Behind the Use of Platelet‐Rich Plasma (PRP) in Scar Management: A Literature Review,” Scars, Burns & Healing 4 (2018): 2059513118808773. 10.1177/2059513118808773 PMC6243404 30479843

[24] E. Nofal , A. Helmy , A. Nofal , R. Alakad , and M. Nasr , “Platelet‐Rich Plasma Versus CROSS Technique With 100% Trichloroacetic Acid Versus Combined Skin Needling and Platelet Rich Plasma in the Treatment of Atrophic Acne Scars: A Comparative Study,” Dermatologic Surgery: Official Publication for American Society for Dermatologic Surgery 40, no. 8 (2014): 864–873. 10.1111/dsu.0000000000000091 25006854

[25] N. Ahramiyanpour , F. Rastaghi , S. Y. Parvar , A. K. Sisakht , S. A. Hosseini , and M. Amani , “Subcision in Acne Scarring: A Review of Clinical Trials,” Journal of Cosmetic Dermatology 22, no. 3 (2023): 744–751. 36315903 10.1111/jocd.15480

[26] M. Kaur , V. K. Sharma , G. Sethuraman , S. Arava , and S. Gupta , “A Split‐Face Randomized Controlled Study Comparing the Efficacy and Safety of Intralesional Radiofrequency‐Assisted Subcision vs Conventional Subcision in Postacne Scars,” Journal of Cosmetic Dermatology 19, no. 5 (2020): 1086–1092. 32233007 10.1111/jocd.13384

[27] N. S. Deshmukh and V. A. Belgaumkar , “Platelet‐Rich Plasma Augments Subcision in Atrophic Acne Scars: A Split‐Face Comparative Study,” Dermatologic Surgery 45, no. 1 (2019): 90–98. 30102625 10.1097/DSS.0000000000001614

[28] A. S. Hassan , M. S. El‐Hawary , H. M. Abdel Raheem , S. H. Abdallah , and M. M. El‐Komy , “Treatment of Atrophic Acne Scars Using Autologous Platelet‐Rich Plasma vs Combined Subcision and Autologous Platelet‐Rich Plasma: A Split‐Face Comparative Study,” Journal of Cosmetic Dermatology 19, no. 2 (2020): 456–461. 31241854 10.1111/jocd.13048

[29] E. Lotfi , M. Shafie'ei , and N. Ahramiyanpour , “Radiofrequency‐Assisted Subcision Combined With Polycaprolactone‐Based Dermal Fillers in the Management of Atrophic Facial Acne Scars: A Pilot Investigative Study,” Skin Research and Technology 28, no. 6 (2022): 865–871. 36321243 10.1111/srt.13228 PMC9907701

[30] A. Nassar , W. El‐Shaarawy , and E. Salah , “Autologous Plasma Gel Injection Combined With Scar Subcision Is a Successful Technique for Atrophic Post‐Acne Scars: A Split‐Face Study,” Journal of Dermatological Treatment 33, no. 2 (2022): 829–835. 32530334 10.1080/09546634.2020.1782322

[31] H. M. Ebrahim , A. Nassar , K. ElKashishy , A. Y. M. Artima , and H. M. Morsi , “A Combined Approach of Subcision With Either Cross‐Linked Hyaluronic Acid or Threads in the Treatment of Atrophic Acne Scars,” Journal of Cosmetic Dermatology 21, no. 8 (2022): 3334–3342. 34927342 10.1111/jocd.14675

[32] B. Kamran , J. Samaneh , D. Maryam , L. Vahideh , A. H. Sima , and G. Hamed , “Subcision for Acne Scarring With and Without Suctioning: A Clinical Trial,” Iranian Journal of Dermatology 14, no. 3 (2011): 95–99.

